# Ly6C^high^ Monocytes Become Alternatively Activated Macrophages in Schistosome Granulomas with Help from CD4+ Cells

**DOI:** 10.1371/journal.ppat.1004080

**Published:** 2014-06-26

**Authors:** Natasha M. Girgis, Uma Mahesh Gundra, Lauren N. Ward, Mynthia Cabrera, Ute Frevert, P'ng Loke

**Affiliations:** Department of Microbiology, New York University School of Medicine, New York, New York, United States of America; University of York, United Kingdom

## Abstract

Alternatively activated macrophages (AAM) that accumulate during chronic T helper 2 inflammatory conditions may arise through proliferation of resident macrophages or recruitment of monocyte-derived cells. Liver granulomas that form around eggs of the helminth parasite *Schistosoma mansoni* require AAM to limit tissue damage. Here, we characterized monocyte and macrophage dynamics in the livers of infected CX3CR1^GFP/+^ mice. CX_3_CR1-GFP^+^ monocytes and macrophages accumulated around eggs and in granulomas during infection and upregulated PD-L2 expression, indicating differentiation into AAM. Intravital imaging of CX_3_CR1-GFP^+^ Ly6C^low^ monocytes revealed alterations in patrolling behavior including arrest around eggs that were not encased in granulomas. Differential labeling of CX_3_CR1-GFP^+^ cells in the blood and the tissue showed CD4^+^ T cell dependent accumulation of PD-L2^+^ CX_3_CR1-GFP^+^ AAM in the tissues as granulomas form. By adoptive transfer of Ly6C^high^ and Ly6C^low^ monocytes into infected mice, we found that AAM originate primarily from transferred Ly6C^high^ monocytes, but that these cells may transition through a Ly6C^low^ state and adopt patrolling behavior in the vasculature. Thus, during chronic helminth infection AAM can arise from recruited Ly6C^high^ monocytes via help from CD4^+^ T cells.

## Author Summary

Macrophages will adopt different characteristics based on different types of inflammatory responses. During infection by parasitic helminths such as *Schistosoma mansoni*, macrophages adopt an “alternatively activated” or M2 phenotype (AAM). These AAM are important for protecting liver hepatocytes from damage caused by the parasite eggs. Here, we examine the cellular source of AAM in the liver of mice infected with *S. mansoni*. We find that AAM during *S. mansoni* infection come from monocytes and not from tissue resident macrophages. Monocytes can be separated into Ly6C^high^ and Ly6C^low^ monocyte subsets. We demonstrate that it is the Ly6C^high^ monocytes that are the precursors of AAM in the liver granulomas, but they might adopt the behavior of Ly6C^low^ monocytes in response to schistosome eggs. Additionally, these Ly6C^High^ monocytes require help from CD4^+^ T cells in order to differentiate into AAM or to maintain this phenotype.

## Introduction

Alternatively activated macrophages (AAM) are a key feature of the immune response elicited by helminth infections [1]. AAM are activated and maintained by the T_H_2 cytokines IL-4 and IL-13 to adopt a phenotype characterized by the expression of signature genes such as arginase-1, Ym1/Chi3l3, Fizz1/Relmα, and PD-L2 [2], [3]. AAM perform distinct functions in different helminth infections. During *Schistosoma mansoni* infection, AAM protect the liver hepatocytes from tissue damage and are critical to organizing the granulomas around the parasite eggs [4]. Without AAM, *S. mansoni* infected mice die from liver and intestinal damage caused by the parasite eggs [4]. In contrast, during *Heligmosoides polygyrus* infection, AAM are important for the expulsion of parasites upon secondary challenge by encasing the parasites in intestinal granulomas [5]. During *Nippostrongylus brasiliensis* infection, AAM are critical for rapidly resolving the acute lung damage caused by migrating larvae [6]. AAM also play an important role in diverse biological functions including metabolic regulation [7], thermoregulation [8], and tumor progression[9].

AAM can be generated through proliferation of resident macrophages or recruitment of inflammatory monocytes [10]. Infection with the tissue-dwelling filarial nematode *Litomosoides sigmodontis* leads to the expansion of AAM mainly through proliferation of tissue-resident macrophages [10], while monocytes have also been described as sources of AAM [11] in models of acute inflammation, including the healing myocardium [12], *Listeria monocytogenes* infection [13] and experimental autoimmune encephalomyelitis [14]. However, it is unclear if populations of AAM that accumulate under conditions of chronic inflammatory stimuli (e.g. in liver granulomas around *S. mansoni* eggs) are derived from recruited monocytes, or through proliferation of resident macrophages.

Two monocyte subsets have been identified through the analysis of knock-in mice with a GFP reporter in the CX_3_CR1 locus [15], [16]. These include the CX_3_CR1^int^ Ly6C^high^ monocytes that rapidly traffic to sites of infection and inflammation and CX_3_CR1^high^ Ly6C^low^ monocytes that patrol blood vessels [13], where they may be responsible for removing cellular debris from the lumen of the vasculature. It has been proposed that CX_3_CR1^int^Ly6C^high^ monocytes preferentially differentiate into inflammatory classically activated macrophages [17]. The developmental relationship between Ly6C^high^ and Ly6C^low^ monocytes has not been fully elucidated. The transcription factor NR4A1 can regulate the differentiation and survival of CX_3_CR1^high^ Ly6C^low^ monocytes directly from macrophage-DC precursors (MDP), suggesting that Ly6C^high^ and Ly6C^low^ monocytes may represent separate lineages [18]. However, CX_3_CR1^int^ Ly6C^high^ monocytes can also differentiate into CX_3_CR1^high^ Ly6C^low^ cells in the bone marrow and blood under steady state conditions [19]–[23]. Hence, there is an active debate over whether CX_3_CR1^high^ Ly6C^low^ monocytes arise through a separate lineage or transition from a CX_3_CR1^int^Ly6C^high^ monocyte state. If AAM arise from recruited Ly6C^low^ monocytes, it is possible that they may require transition from a Ly6C^high^ monocyte state.

Although AAM have been shown to protect the host from pathology during *S. mansoni* infection [24], [25], it remains unclear whether granuloma AAM accumulate through IL-4 driven proliferation or through recruitment of monocyte-derived macrophages. The focus of our studies is to determine the origin of AAM that accumulate in egg granulomas. We used the *Cx3cr1* ^gfp/+^ mice [16] to investigate the dynamics of AAM accumulation into the liver granulomas of *S. mansoni* infected mice. By intravital imaging, we find marked alterations in the dynamics of cellular behavior for CX_3_CR1-GFP+ cells in the liver granulomas. By using a combination of differential blood and tissue antibody labeling, adoptive monocyte transfers, and EdU pulsing we find that the AAM in liver granulomas may arise predominantly from recruited Ly6C^high^ monocytes, but that these cells may transition through a Ly6C^low^ state.

## Results

### CX_3_CR1-GFP^+^ cells accumulate in the liver when *S. mansoni* eggs appear without proliferation

**Figure 1:**
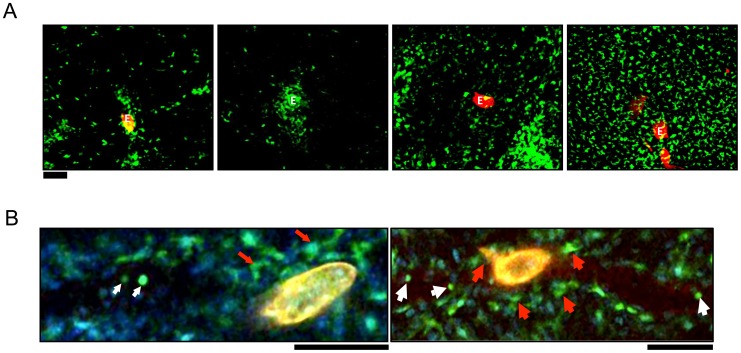
CX_3_CR1-GFP^+^ cells accumulate in the hepatic granulomas around *S. mansoni* eggs. (**A**) Intravital confocal imaging of liver granulomas in *S. mansoni*-infected CX_3_CR1^GFP/+^ mice between 6–8 weeks post-infection Eggs visualized through auto-fluorescence can be seen in the red channel and are labeled “E.” CX_3_CR1-GFP+ cells are shown in green. Scale bar = 100 µm. (**B**) Intravital snapshot of an infected CX_3_CR1^GFP/+^ mouse showing stationary cells with macrophage-like morphology (red arrows) around a schistosome egg (yellow) and motile intravascular GFP^+^ cells (white arrows).

AAM recruited to liver granulomas following *S. mansoni* infection are CX_3_CR1-GFP^+^ using *Cx3cr1* ^gfp/+^ reporter mice at seven weeks post-infection [26]. Using confocal microscopy, we observed that CX_3_CR1-GFP^+^ cells with extended processes and macrophage-like morphology are found within granulomas at various stages of granuloma formation (Figure 1A). In small granulomas, CX_3_CR1-GFP^+^ cells are directly in contact with the eggs (left most panels), but within larger granulomas the CX_3_CR1-GFP^+^ cells are found in the fringes of the granuloma (second to rightmost panels), or dispersed throughout the liver (rightmost panels). Because the *Cx3cr1* ^gfp/+^ reporter mice have been used extensively to track monocyte differentiation [15], [19] we hypothesized that the CX_3_CR1-GFP^+^ cells in the granulomas expressing markers of AAM [26] are derived from monocytes, rather than through proliferation of tissue resident macrophages [10].

Male and female schistosomes mature and pair before the female parasites start producing eggs approximately 5 weeks post-infection. To examine the earliest stages of granuloma formation, we performed confocal microscopy of infected livers at 5 weeks post-infection, when eggs are just beginning to be lodged in the liver (Figure 1B). In *S. mansoni* infected mice, we observed round monocyte-like CX_3_CR1-GFP^+^ cells (white arrows) inside the blood vessels near schistosome eggs. However, CX_3_CR1-GFP^+^ cells directly in contact with eggs had extended membrane processes (red arrows), suggesting that encounter with schistosome eggs may differentiate CX_3_CR1-GFP^+^ monocytes into macrophages.

**Figure 2:**
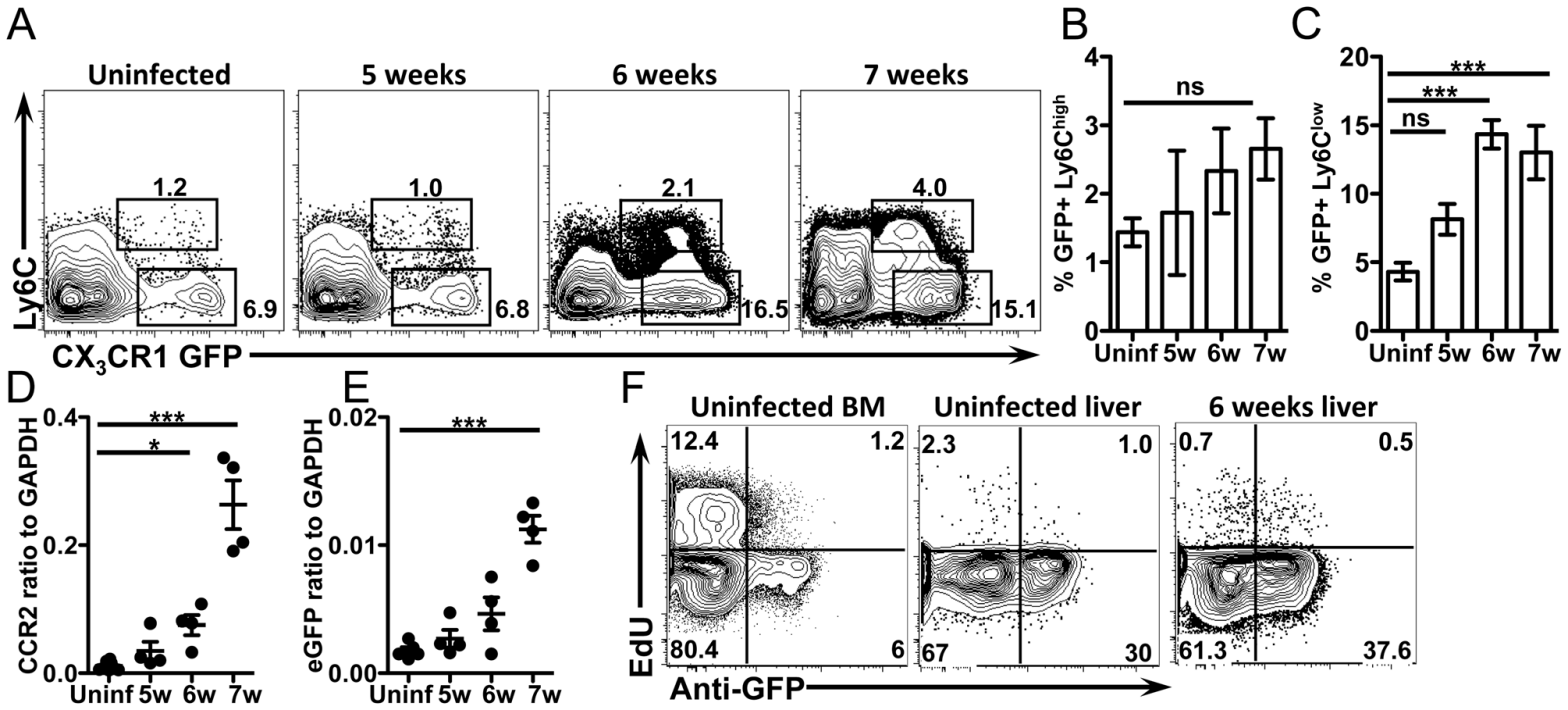
CX_3_CR1-GFP^+^ monocytes accumulate in the liver when *S. mansoni* eggs appear without proliferation. (**A–C**) Representative flow cytometry plots and graphs display the proportion of GFP^+^ Ly6C^high^ and Ly6C^low^ monocytes from CX_3_CR1^GFP/+^ mice gated on live, single, lineage (CD3, B220, DX5 and Siglec F) negative, SSC^low^ cells. (**D–E**) qRT-PCR analysis of CCR2 and eGFP expression in the liver. (**F**) Representative contour plots show *in situ* EdU labeling of liver leukocytes gated on live, single, lineage negative, CD11b^+^ cells. Results are representative of two experiments that included n = 3–4 mice/group. Statistical significance was determined by One-way ANOVA with Dunnett's test. * = p<0.05, ** = p<0.001, *** = p<0.0001.

To investigate differences in CX_3_CR1-GFP^+^ monocyte recruitment before and after granulomas begin to form around eggs (i.e. 5 weeks vs. 6 and 7 weeks) we compared uninfected mice, and mice infected for 5, 6 or 7 weeks. Flow cytometric analysis of liver leukocytes showed an increase in the frequency of both Ly6C^high^ and Ly6C^low^ CX_3_CR1-GFP^+^ monocytes in infected mice at 6 and 7 weeks, which coincides with the formation of granulomas in the liver (Figure 2A, 2B and 2C). Quantitative real-time PCR (qRT-PCR) analysis of liver tissue confirmed a significant increase in both CCR2 (Figure 2D), which is expressed on Ly6C^high^ monocytes [15], [27], and eGFP (Figure 2E) expression in infected mice after granuloma formation, confirming that inflammatory monocytes accumulate in the liver when granulomas are formed.

Because AAM can also accumulate through proliferation [10] we next determined if CX_3_CR1-GFP^+^ cells in the liver also proliferate *in situ*. Uninfected and infected mice were pulse labeled with the nucleoside analog EdU for 3 hours to quantify the proportion of cells that were in S phase at the time of sacrifice [10]. The frequency of cells that were in S phase was the same between uninfected mice and mice at 6 weeks post-infection (Figure 2F), indicating the CX_3_CR1-GFP^+^ cells are not proliferating more at this time point during infection. As a positive control, EdU incorporation into CD11b+ cells from the bone marrow was assayed in the same experiment (Figure 2F). Together, these data suggest that the CX_3_CR1-GFP^+^ AAM incorporated into the hepatic granulomas are primarily derived from recruitment of monocytes rather than through proliferation of tissue resident macrophages.

### The presence of *S. mansoni* eggs alters the patrolling behavior of CX_3_CR1-GFP^+^ cells in the liver sinusoids

**Figure 3:**
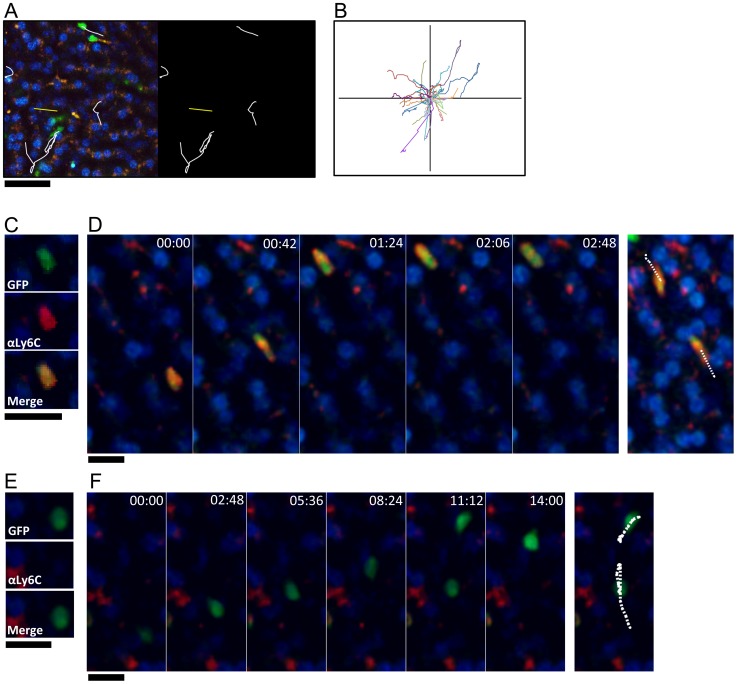
GFP^+^Ly6C^low^ monocytes exhibit patrolling behavior, while GFP^+^Ly6C^high^ cells transit rapidly through the sinusoids. (**A**) High magnification snapshot showing tracks of GFP+ crawling cells (white) and GFP+ cells that move rapidly through the sinusoids (yellow) in a steady state uninfected liver. Scale bar = 50 µm. (**B**) Representative tracks of crawling monocytes in steady state uninfected livers. (**C and E**) Intravital confocal microscopy of an uninfected *Cx_3_cr1^gfp/+^* mouse showing GFP+ (green) monocytes that are either Ly6C^high^ (**C**) or Ly6C^low^ (**E**) after intravenous anti-Ly6C Ab staining (red). (**D and F**) Time-lapse images from an uninfected *Cx_3_cr1^gfp/+^* mouse injected with anti-Ly6C Ab showing two GFP+ Ly6C^high^ monocytes (**D**) and two GFP+ Ly6C^low^ monocytes migrating through the sinusoids (**F**). GFP is shown in green, anti-Ly6C in red, and nuclei in blue. Snapshots were taken in single z planes. Scale bars = 20 µm. Tracks are shown in white.

CX3CR1-GFP+ monocytes that “patrol” the luminal side of blood vessels have been observed by intravital microscopy in mesenteric vessels [13] and the vascular network of the kidney cortex [28]. We used intravital microscopy to track the behavior of CX_3_CR1-GFP^+^ cells within liver sinusoidal vessels and tissues *in vivo*. In the liver sinusoids of uninfected animals at steady state, we observed CX_3_CR1-GFP^+^ cells that crawled along the sinusoidal vessels (Figure 3A, Movie S1 and Movie S2) with complex tracks (Figure 3B) characteristic of the patrolling behavior that has been described for Ly6C^low^ monocytes [13], [28]. We also observed CX_3_CR1-GFP^+^ cells that transited rapidly through the sinusoids and had shorter, less complex tracks (Figure 3A). When we intravenously (i.v.) injected fluorescently labeled anti-Ly6C antibody [28], prior to intravital imaging, we found that CX_3_CR1-GFP^+^ cells that were brightly labeled with anti-Ly6C (Figure 3C) did not exhibit crawling behavior (Figure 3D), whereas CX_3_CR1-GFP^+^ cells the were not labeled by anti-Ly6C (Figure 3E) exhibited crawling behavior (Figure 3F). This is consistent with the GFP+ cells with crawling behavior in the liver sinusoids being Ly6C^low^ monocytes. Our observations are also consistent with the behavior of Ly6C^low^ monocytes in the mesenteric vessels [13] and kidney cortex [28], whereby the direction of crawling movement was independent of the direction of blood flow, with an instantaneous velocity of 2 to 21 µm/min (7 µm/min on average).

**Figure 4:**
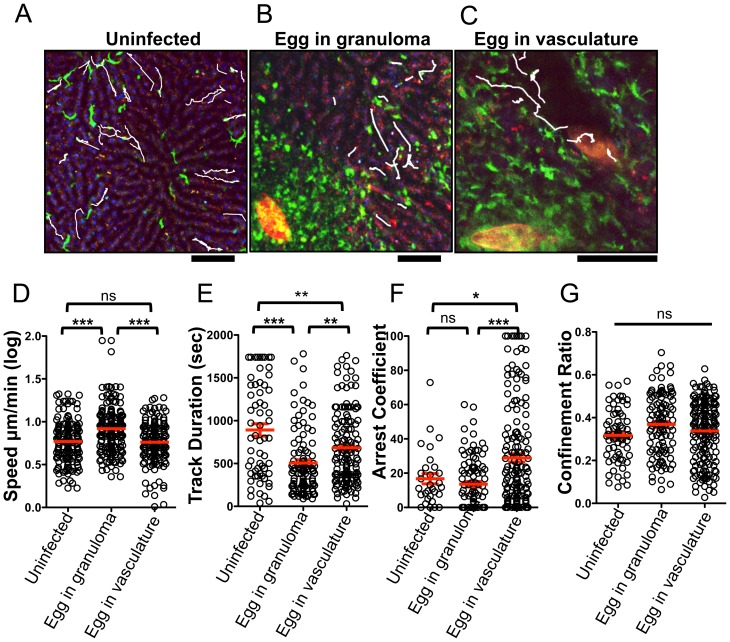
The presence of *S. mansoni* eggs alters the patrolling behavior of CX_3_CR1-GFP^+^ monocytes in the liver sinusoids. (**A–C**) Maximum projections of Z stacks from the livers of (**A**) uninfected *Cx_3_cr1^gfp/+^* mice and *S. mansoni-*infected *Cx_3_cr1^gfp/+^* mice with an egg encapsulated in a granuloma (**B**), or with an egg in the blood vessels (**C**). Sinusoidal vessels (dark areas) and tissue architecture are visualized by nuclear staining (blue) and auto-fluorescence in the red channel. Parasite eggs are auto-fluorescent and can be seen in red. White tracks showing the paths of individual motile CX_3_CR1-GFP^+^ cells (green) are overlayed onto images. Scale bars = 50 µm (**D**) Log-transformed mean speed (µm/min), (**E**) track duration of motile GFP^+^ cells, (**F**) arrest coefficient (fraction of time cell crawls <2 µm/min) and (**G**) confinement ratio (Displacement/track length). Motility data is pooled from 3 mice for uninfected mice (n = 68 cells), 5 mice for fully developed granulomas (n = 182 cells). Data for exposed eggs in the vasculature is pooled from 6 mice (n = 143). Scale bars = 100 µm. * = p<0.05, ** = p<0.001, *** = p<0.0001.

We next examined if deposition of schistosome eggs alters the behavior of CX_3_CR1-GFP^+^ cells (Figure 4). At 8 weeks post-infection some of the eggs are completely encapsulated in fully developed granulomas (Figure 4B), whereas other eggs are lodged in the blood vessels (Figure 4C, Movie S3), presumably because they were more recently released by the female parasites. The round CX_3_CR1-GFP^+^ cells inside the sinusoids of *S. mansoni* infected mice were motile with patrolling behavior, whereas the CX_3_CR1-GFP^+^ cells within granulomas had extended membrane processes and macrophage-like morphology and were sessile (Movie S3). Compared to uninfected animals (Figure 4A, Movie S2), crawling CX_3_CR1-GFP^+^ cells in the vasculature near fully developed egg granulomas (Figure 4B, Movie S4) exhibited a significant increase in speed (Figure 4D), along with a decrease in track duration (Figure 4E) suggesting that proximity to an egg granuloma can increase the motility of crawling CX_3_CR1-GFP^+^ cells. In contrast, crawling CX_3_CR1-GFP^+^ cells near eggs that are lodged in the blood vessels (but have not been encapsulated in a granuloma) (Figure 4C, Movie S3 and S5) exhibited a significant reduction in speed (Figure 4D), an increase in track duration (Figure 4E), and increase in arrest coefficient (Figure 4F). The retention of crawling monocytes in response to the exposed eggs is consistent with the retention of Ly6C^low^ monocytes in the kidney capillaries after TLR7 mediated inflammation [28]. The confinement ratio of the crawling monocytes is unaltered in response to granuloma formation (Figure 4G) indicating that the monocytes maintain patrolling behavior regardless of egg exposure. Hence, the crawling CX_3_CR1-GFP^+^ cells may be retained in the sinusoids in response to exposed eggs because the cells are sensing secreted products from the eggs or vascular damage, but these changes in cellular behavior are no longer observed when the eggs become encased in granulomas.

### CX_3_CR1-GFP^+^ cells upregulate PD-L2 as they extravasate and accumulate in the liver

**Figure 5:**
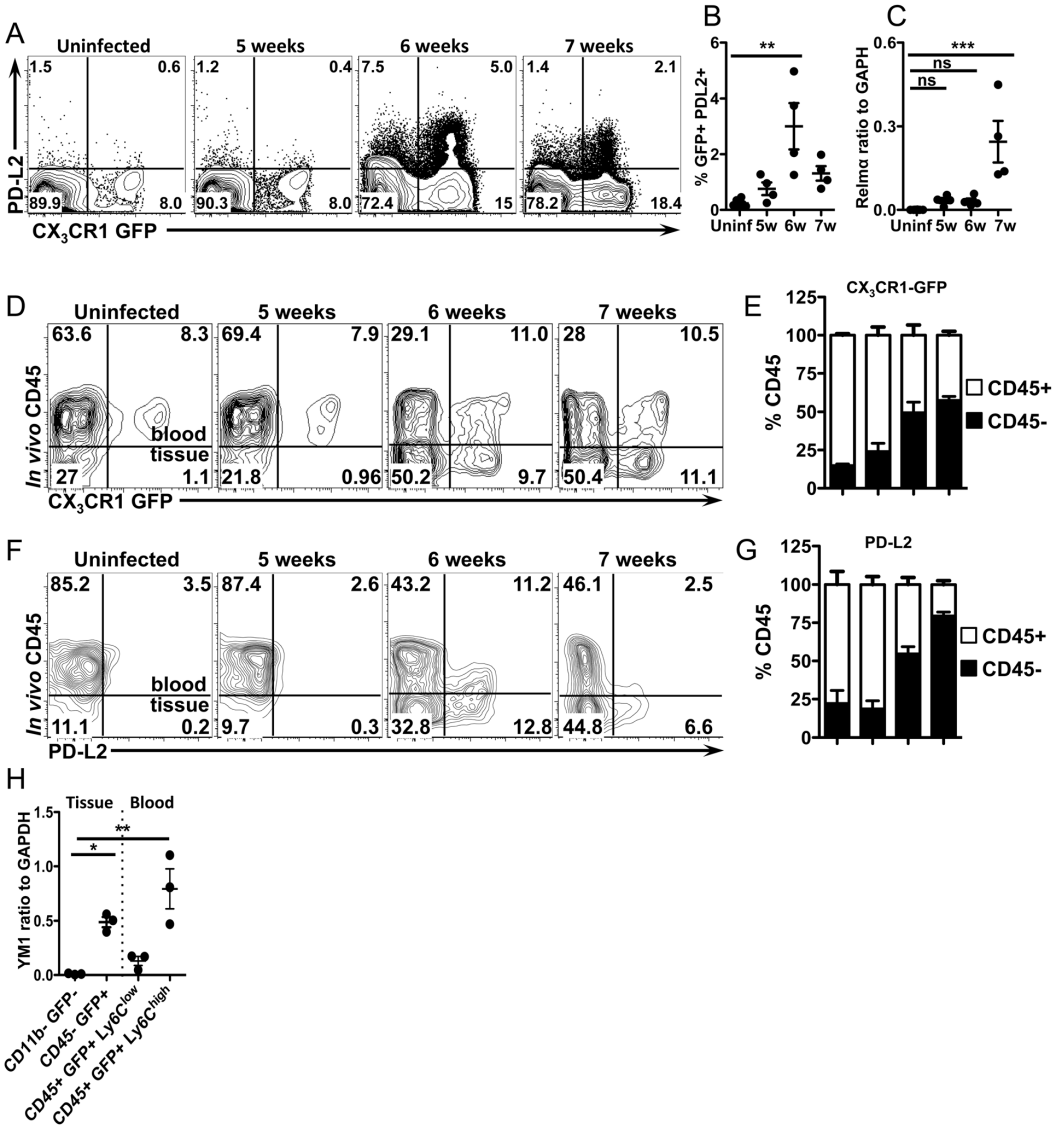
CX_3_CR1-GFP^+^ cells upregulate PD-L2 as they extravasate and accumulate in liver tissue. (**A and B**) Representative contour plots and graphs displaying PD-L2 expression on liver leukocytes isolated from uninfected or infected *CX_3_CR1^gfp/+^* mice gated on live, single, lineage (CD3, B220, DX5 and Siglec F) negative, SSC^low^ cells. n = 4 mice/group. (**C**) qRT-PCR analysis of Relmα expression. (**D**) Representative contour plots show blood/tissue partitioning of GFP^+^ liver leukocytes from CX_3_CR1^GFP/+^ mice using *in vivo* CD45 staining. Plots display cells gated on single, live, lineage negative cells. (**E**) The proportion of CD45^+^ (white) or CD45^−^ (black) cells gated on live single, lineage^−^, GFP^+^ cells. (**F**) Contour plots display blood/tissue partitioning of PDL2^+^, GFP^+^ cells gated on single, live, lin-, CX_3_CR1-GFP^+^ cells. (**G**) Proportions of GFP^+^PDL2^+^ cells that are CD45^+^ (white) or CD45^−^ (black) as in (**E**). (**H**). qRT-PCR analysis of YM1 expression in liver leukocytes sorted from 8 week infected mice using *in vivo* CD45 staining to isolate CD45−(tissue) GFP+ granuloma macrophages from CD45+ (blood) GFP+, Ly6C^high^ and Ly6C^low^ monocytes. CD11b-GFP− cells from infected mice were used as a negative control. Dots represent individual mice from one of two experiments. Statistical significance was determined by One-way ANOVA with Dunnett's test. ** = p<0.001, *** = p<0.0001. Results are representative of at least two experiments that include n = 3–4 mice/group.

We previously showed that the costimulatory ligand PD-L2 is upregulated on AAM [29], hence we determined if the accumulating CX_3_CR1-GFP^+^ cells express PD-L2. Indeed the frequency of PD-L2 expressing CX_3_CR1-GFP^+^ cells in the liver peaks at 6 weeks post-infection (Figure 5A and 5B), providing us with a surface marker to determine when CX_3_CR1-GFP^+^ cells begin to adopt the AAM phenotype. The expression of *Relmα*, a different marker for AAM, has slightly different kinetics and is higher at 7 weeks post-infection (Figure 5C), which may be due in part to increased RELMα production by eosinophils during *S. mansoni* infection [6].

To determine if CX_3_CR1-GFP^+^ cells become alternatively activated as they accumulate in the tissue and are no longer directly exposed to the liver sinusoids, we used *in vivo* CD45 staining, which has recently been shown to allow discrimination of intravascular and extravascular leukocytes [30]. Mice were injected i.v. with fluorescently labeled antibodies to CD45 immediately before sacrifice, which labels leukocytes in the blood, but not those in the tissue [30], enabling us to distinguish if CX_3_CR1-GFP^+^ cells are intravascular (CD45^+^) or extravascular (CD45^−^) (Figure 5D). In both uninfected mice and infected mice that do not yet have established granulomas (5 weeks post-infection), 80–90% of the CX_3_CR1-GFP^+^ cells were CD45^+^ and only 10–20% are CD45^−^ (Figure 5D and 5E), indicating that they are mostly intravascular when granulomas are not present. Consistent with our imaging data, for infected mice with granulomas (6 and 7 weeks post-infection), 50–60% of the GFP^+^ cells are CD45^−^ (Figure 5D and 5E), indicating that they have extravasated into the tissues.

We then determined when these cells adopt the phenotype of AAM by staining for PD-L2 (Figure 5F and 5G). In the absence of granulomas at 5 weeks post-infection and in uninfected mice there are very few PD-L2^+^ cells (Figure 5F). At 6 weeks post-infection, 50% of GFP^+^, PD-L2^+^ cells were intravascular, while 50% were in the tissue (Figure 5F and 5G). However, by 7 weeks post-infection, 80% of GFP^+^, PD-L2^+^ cells were CD45- (Figure 5F and 5G). Together these results show that CX_3_CR1-GFP^+^ cells begin to adopt AAM phenotype intravascularly and then these cells extravasate and accumulate over time in the extravascular tissues. To confirm the results from PD-L2 staining, we FACs sorted cells after *in vivo* CD45 staining from infected mice for RT-PCR analysis with other AAM markers (Figure 5H, Figure S1). Consistent with FACS analysis, GFP+ cells in the tissue (CD45−) express YM1/Chi3l3 much more than GFP- cells in the tissue. Surprisingly, Ly6C^high^ monocytes in the blood express more Ym1/Chi3l3 than Ly6C^low^ monocytes (Figure 5H). We originally expected that the GFP+ cells that were encountering eggs in the vasculature (and perhaps becoming alternatively activated) would be Ly6C^low^ monocytes because of their crawling behavior (Figure 4).

### Ly6C^high^ monocytes extravasate more efficiently than Ly6C^low^ monocytes while reducing Ly6C expression and upregulating PD-L2

**Figure 6:**
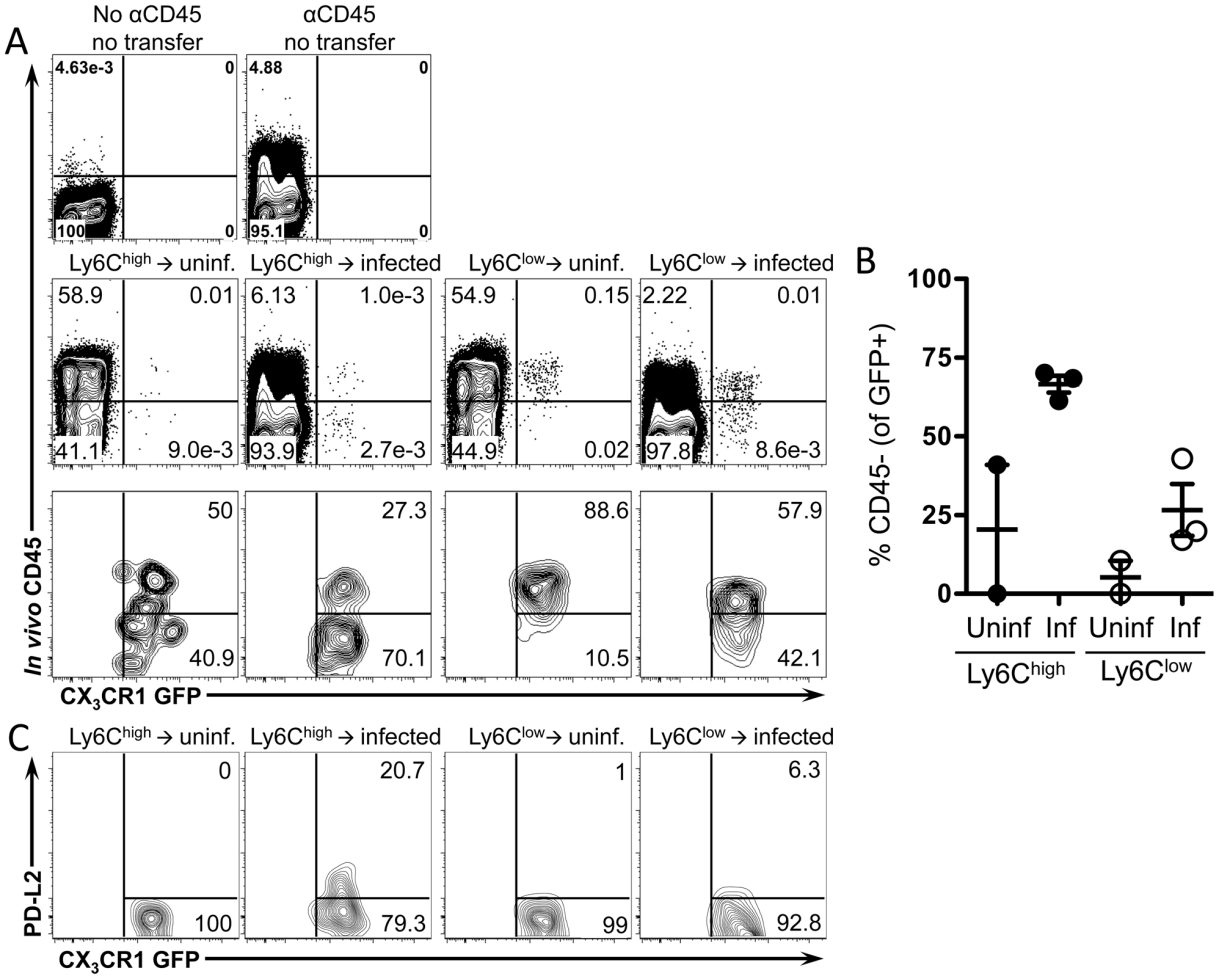
Ly6C^high^ monocytes extravasate more efficiently than Ly6C^low^ monocytes and upregulate PD-L2. Splenic monocytes were purified from *CX_3_CR1^GFP/+^* mice. Splenocytes depleted of CD19^+^ and CD3^+^ cells were FACS sorted to isolate single, live, CD3−, B220^−^, Ly6G^−^, MHCII^−^, DX5^−^, Siglec F^−^, CD11c^−^, F4/80^−^, CD11b^+^, GFP^+^, Ly6C^high^ or Ly6C^low^ monocytes. Transferred monocytes were recovered from the livers of uninfected or 8 week-infected C57BL/6 recipient mice 24 hours after transfer and analyzed by flow cytometry for blood/tissue partitioning of GFP^+^ cells using *in vivo* CD45 staining. (**A**) Representative plots show live, single, lin^−^, CD11b^+^ cells. Lower panels in (**A**) are also gated on transferred GFP+ cells. (**B**) The graph displays the proportion of transferred GFP^+^ cells that entered the tissue (CD45^−^) and includes mice (n = 3) pooled from two independent experiments. (**C**) Contour plots display PD-L2 expression on transferred cells gated on live, single, lin^−^, CD11b^+^, GFP^+^ cells.

We next determined whether the alternatively activated CX_3_CR1-GFP^+^ macrophages in the liver granulomas are derived from Ly6C^high^ or Ly6C^low^ monocytes. We initially hypothesized that Ly6C^low^ monocytes may be the precursors of CX_3_CR1-GFP^+^ AAM since we observed GFP+ cells with behavior characteristic of Ly6C^low^ monocytes accumulating in the liver sinusoids around schistosome eggs (Figure 4). However, Ly6C^high^ monocytes in the blood express more Ym1/Chi3l3 than Ly6C^low^ monocytes (Figure 5H). Hence, to determine which cells are the precursors of granuloma AAM, we adoptively transferred FACs sorted pure populations of CX_3_CR1-GFP^+^ Ly6C^high^ or Ly6C^low^ monocytes (Figure S2) isolated from the spleens of *Cx_3_cr1^gfp/^* ^+^ mice into uninfected or infected C57BL/6 recipient mice (Figure 6). Using *in vivo* CD45 labeling to analyze blood/tissue partitioning, 24 hours after transfer, both Ly6C^high^ and Ly6C^low^ GFP^+^ cells could be detected in the livers of uninfected and infected recipient mice (Figure 6A). In uninfected mice that received Ly6C^high^ monocytes, very few transferred cells can be recovered from the liver (Figure 6A), which is consistent with the natural tendency for Ly6C^high^ monocytes to home to the bone marrow in the absence of inflammation [19]. In contrast, more Ly6C^low^ monocytes can be recovered from the livers of uninfected mice (Figure 6A), which is consistent with the vascular patrolling behavior of these cells [13]. However, blood/tissue partitioning by *in vivo* CD45 labeling showed that the Ly6C^low^ monocytes remained in the vasculature of uninfected mice (Figure 6A and 6B). In contrast, in infected mice ∼70% of Ly6C^high^ monocytes and ∼25% of Ly6C^low^ monocytes were able to enter the tissue (Figure 6A and 6B), hence Ly6C^high^ monocytes enter tissue more efficiently than Ly6C^low^ monocytes.

To determine if the transferred Ly6C^high^ and Ly6C^low^ monocytes differentiate into AAM, we examined upregulation of PD-L2 on transferred GFP^+^ cells. Only transferred Ly6C^high^ monocytes upregulate PD-L2 (Figure 6E), suggesting that only transferred Ly6C^high^ monocytes have adopted an AAM phenotype. Hence, Ly6C^high^ monocytes are likely precursors of the CX3CR1-GFP^+^, PD-L2^+^ cells that accumulate in the liver during granuloma formation.

### Ly6C^high^ monocytes reduce Ly6C expression, exhibit crawling behavior, and upregulate markers of alternative activation after infection

**Figure 7:**
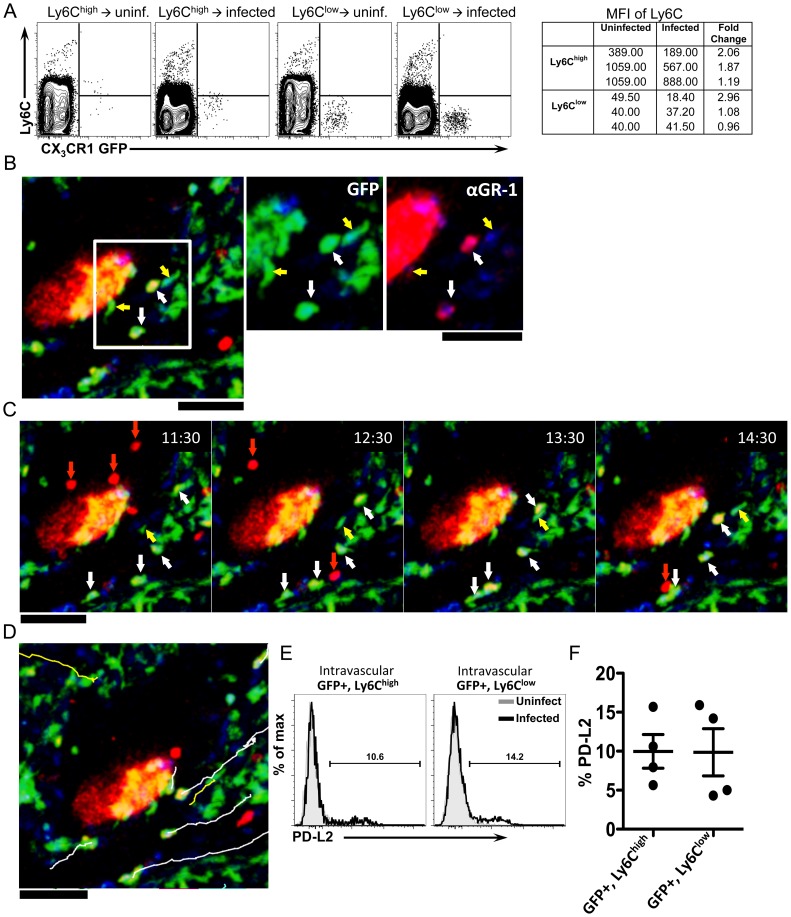
Ly6C^high^ monocytes reduce Ly6C expression, exhibit crawling behavior, and upregulate markers of alternative activation after infection. (**A**) Contour plots display Ly6C expression on monocytes isolated from *CX_3_CR1^GFP/+^* mice and transferred into uninfected or infected C57BL/6 mice (as described in Figure 6). Lines serve as guides to show the level of Ly6C expression. (**B**) Intravital confocal microscopy of an infected *Cx_3_cr1^gfp/+^* mouse showing crawling GFP+ cells (green) that are either Ly6C^+^ (white arrows) or Ly6C^−^ (yellow arrows) after intravenous anti-GR-1 (Ly6C/G) Ab labeling (red). (**C**) Time-lapse images from an 8-week infected *Cx_3_cr1^gfp/+^* mouse injected with anti-Ly6C/Ly6G Ab showing the movement of GFP+ Ly6C+ cells (white arrows), GFP+ Ly6C− cells (yellow arrows) and GFP− GR-1+ neutrophils (red arrows). (**D**) Tracks of GFP+ Ly6C+ cells (white lines), GFP+ Ly6C− cells (yellow lines) shown in the time lapse. GFP is shown in green, anti-Ly6C (GR-1) in red, and nuclei in blue. Scale bars = 50 µm. Data is representative of experiments from 2 mice. (**E–F**) Representative histograms (**E**) and graph (**F**) display the percentage of PD-L2+GFP+Ly6Chigh and PD-L2+GFP+Ly6Clow cells from the blood (by *in vivo* CD45 staining) of 6-week infected mice (black) or uninfected mice (gray). Cells are gated on single, live, lineage negative, *in vivo* CD45+, GFP+ cells. Data is representative of two independent experiments. N = 4 mice/group.

It has been suggested that Ly6C^low^ monocytes are derived from Ly6C^high^ monocytes [20]–[24]. Although the monocyte transfer experiments suggest that Ly6C^high^ monocytes serve as precursors of granuloma AAM, they may transition through a Ly6C^low^ intermediate state. When we examined Ly6C expression on transferred GFP^+^ monocytes (Figure 7A), Ly6C^high^ CX^3^CR1-GFP^+^ cells transferred into infected mice exhibited a 1.7-fold reduction in the mean fluorescence intensity of Ly6C, compared to cells transferred into uninfected mice (Figure 7A). In mice that received Ly6C^low^ monocytes, Ly6C expression remained low (Figure 7A). This suggests that Ly6C^high^ monocytes may transition through a Ly6C^low^ intermediate state in infected livers, but not in naïve uninfected mice.

We had observed through the i.v. delivery of fluorescently labeled anti-Ly6C/Ly6G antibody just prior to intravital imaging, that in naïve uninfected mice, CX_3_CR1-GFP^+^ cells in the liver sinusoids that were brightly labeled with anti-Ly6C (Figure 3C) did not exhibit crawling behavior (Figure 3C and 3D). We next used this same approach to determine if some of the crawling GFP+ cells in infected mice are Ly6C^high^. In contrast to naïve mice, with mice infected for 8 weeks we could observe by intravital microscopy both GFP+, Ly6C− cells (Figure 7B, yellow arrows), as well as GFP+, Ly6C+ cells Figure 7, white arrows) that exhibit crawling behavior along the vessels near an exposed schistosome egg (Figure 7C and 7D and Movie S6). There are also some motile antibody-labeled cells that are GFP−, which are likely to be neutrophils (Figure 7C, red arrows). Hence, Ly6C+ monocytes can be observed to adopt patrolling behavior in *S. mansoni* infected mice (but not naïve uninfected mice). Thus, the GFP+ cell populations characterized by intravital microscopy (Figure 4) are likely comprised of both Ly6C^high^ and Ly6C^low^ monocytes. These results suggest that during an inflammatory response, inflammatory Ly6C^high^ monocytes recruited into the blood vessels may adopt the patrolling behavior of Ly6C^low^ monocytes.

Although we find that only transferred Ly6C^high^ monocytes upregulate PD-L2 (Figure 6C) in infected mice, they may have adopted Ly6C^low^ monocyte behavior as they encounter schistosome eggs in the vasculature. We can label cells in the vasculature by *in vivo* CD45 staining (Figure 5). We gated on CD45+ (Blood) Ly6C^high^ CX3CR1-GFP+ monocytes, as well as CD45+ (Blood) Ly6C^low^ CX3CR1-GFP+ monocytes, to examine PD-L2 expression on these cells at 6 weeks post-infection, when PD-L2+ cells can be found in the blood (Figure 5F). We find that ∼10% of both GFP+, Ly6C^high^ and GFP+, Ly6C^low^ cells in the blood expressed PD-L2 (Figure 7E and F). One possibility might be that Ly6C^low^ monocytes that are PD-L2+ may have originally been Ly6C^high^.

### CD4+ T cells are required for upregulation of PD-L2 and alternative activation

**Figure 8:**
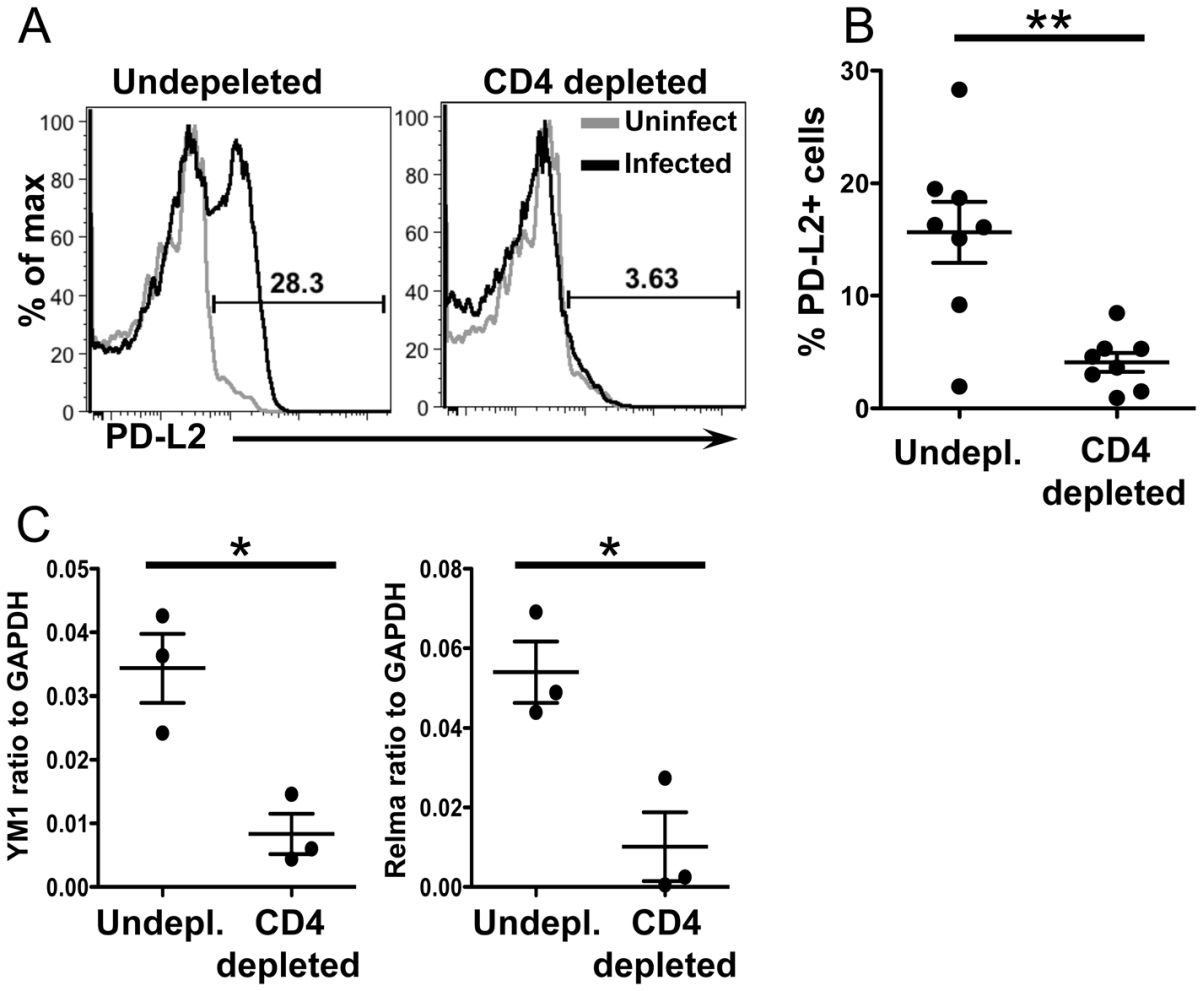
CD4+ T cells are required for upregulation of PD-L2 and alternative activation. (**A**) Infected mice were depleted of CD4^+^ T cells from 5.5–6.5 weeks post-infection. Representative histogram plots display PD-L2 expression on live, single, lin−, CD11b^+^ GFP^+^ cells. (**B**) Percentage of PD^−^L2^+^ cells in undepleted and depleted mice gated as in (**A**). (**C**) qRT-PCR analysis of YM1 and Relmαexpression in whole tissue. CD4 depletion results are pooled from two independent experiments for a total of n = 8. qRT-PCR results are from a single experiment. Error bars represent SEM and statistical significance was determined using the Mann-Whitney test. * = p<0.05, ** = p<0.001.

CD4^+^ T cell help is required for AAM during chronic helminth infection, but not in acute wound healing models of alternative activation [31]. To determine if the accumulation of PD-L2^+^ AAM from CX_3_CR1-GFP^+^ monocytes was CD4^+^ T cell dependent, we depleted CD4^+^ T cells from 5.5 to 6.5 weeks post-infection and analyzed PD-L2 expression on CX_3_CR1-GFP^+^ cells from the liver (Figure 8). CD4^+^ T cell depletion led to a modest increase in the frequency of Ly6C^high^ monocytes in the liver, but this difference was not significant (data not shown). However, CD4 depletion significantly reduced the frequency of PD-L2^+^ CX_3_CR1-GFP^+^ cells (Figure 8A and 8B). CD4+T cell depletion also reduced levels of the AAM markers *Relmα* and *Chi313* in the liver tissue (Figure 8C). Hence, CD4^+^ T cells are required for the accumulation of PD-L2+ AAM from CX_3_CR1-GFP^+^ cells in the liver granulomas.

## Discussion

The *S. mansoni* egg granuloma requires the reorganization of various immune cell types in the affected liver into a structure that protects the liver hepatocytes from further tissue damage [32]. AAM play a critical role in the organization of the granulomas and their absence leads to a fatal outcome [4]. In this study we find that these AAM likely originate from inflammatory Ly6C^high^ monocytes from the blood.

Our intravital imaging data demonstrates that the granulomas consist of a network of sessile CX_3_CR1-GFP^+^ macrophages with almost no cellular displacement, analogous to the mycobacterial granuloma [33]. In contrast, the sinusoidal blood vessels support the patrolling movement of round CX_3_CR1-GFP^+^ cells characteristic of Ly6C^low^ monocytes, which have been suggested to be precursors of AAM [13]. These Ly6C^low^ monocytes have recently been shown to accumulate at sites of vascular damage in the kidney capillaries in response to TLR7-dependent signals [28]. Around schistosome eggs that are lodged in the liver sinusoids, these CX_3_CR1-GFP+ cells exhibited altered patrolling behavior consistent with these cells being retained in the vasculature [28]. However, patrolling behavior around eggs in fully developed granulomas was not altered, suggesting that CX_3_CR1-GFP^+^ crawling monocytes respond to eggs when they are in the blood, but not when they are encased in granulomas.

It is important to note that other cell types express CX_3_CR1-GFP^+^. CD4^+^ T cells represent approximately 10% of the CX_3_CR1-GFP^+^ population at 7 weeks post-infection (data not shown). While we could image *Rag_2_* ^−/−^, γ*c* ^−/−^, *Cx3cr1^gfp/+^* mice, in which monocytes are the only blood cells expressing GFP, these mice were not available and *S. mansoni* does not develop normally in these mice [34]. Additionally, T cells and NK cells have very different morphology and none of these other cell types have been described to exhibit the slow crawling/patrolling behavior, hence we are confident that the cells we are tracking are predominantly monocytes.

After adoptive transfer into *S. mansoni* infected mice, Ly6C^high^ monocytes extravasated more efficiently than Ly6C^low^ monocytes into the liver tissues and upregulated PD-L2. This result suggested that the Ly6C^high^ monocytes are the precursors of the granuloma CX_3_CR1-GFP^+^ AAM. This was rather unexpected to us because by intravital microscopy we had observed that the motile round CX_3_CR1-GFP^+^ monocytes that encounter eggs in the liver exhibited crawling behavior that is characteristic of Ly6C^low^ monocytes. However, when we used intravital microscopy in combination with *in vivo* staining using anti-Ly6C/G antibodies to visualize Ly6C/G+ cells in the liver, we found that CX_3_CR1-GFP^+^ cells crawling in the sinusoidal vessels of infected mice are comprised of both GFP+Ly6C^high^ and GFP+, Ly6C^low^ cells. Further, when we isolated GFP+Ly6C^high^ and GFP+ Ly6C^low^ monocytes from the sinusoidal vessels of infected mice (by *in vivo* CD45 staining), only GFP+, Ly6C^high^ monocytes expressed the AAM marker YM1.

Two different models may explain our results. One possibility is that Ly6C^high^ monocytes are recruited to the liver sinusoids and then differentiate into Ly6C^low^ monocytes with patrolling behavior and then subsequently into AAM in the tissues. This hypothesis is consistent with several studies that have shown that Ly6C^high^ monocytes can become Ly6C^low^ monocytes [19]–[22], [35], including the CX_3_CR1-GFP+ cells that accumulate in the intestinal tract in response to inflammation [27]. We observed that transferred Ly6C^high^ monocytes had reduced expression of Ly6C. By intravital imaging, we also found that although CX_3_CR1-GFP+ Ly6C^high^ cells move rapidly through the livers of uninfected mice, the CX_3_CR1-GFP+ cells crawling in the sinusoids of infected mice were a mixture of Ly6C^high^ and Ly6C^low^ cells. This suggests that Ly6C^high^ monocytes adopt crawling behavior in response to infection and supports the hypothesis that Ly6C^high^ monocytes may transition through a Ly6C^low^ state as they are differentiating into AAM.

Another possibility is that Ly6C^high^ monocytes directly differentiate into AAM, and the accumulation of CX_3_CR1-GFP+ cells Ly6C^high^ and Ly6C^low^ cells in the liver sinusoids have distinct functions. The biological function of Ly6C^low^ monocytes and their differentiation properties are unclear. Recently, they were shown to scan capillaries and remove cellular debris and microparticles as “housekeepers” of the vasculature [28]. Since schistosome eggs are too large to be removed by the Ly6C^low^ monocytes, they may be retained in the vasculature to provide early signals for the formation of a granuloma. When eggs are completely encapsulated into granulomas and are sequestered away from the vasculature, they no longer influence the patrolling behavior of Ly6C^low^ monocytes in the sinusoids. In future experiments, the *S. mansoni* model could be used to investigate how Ly6C^low^ monocytes respond to a dangerous foreign body in the vasculature that is too large to be phagocytosed.

We showed that CD4^+^ helper cells are needed to induce the accumulation of PD-L2+ CX_3_CR1-GFP+ AAM in the liver granulomas of *S. mansoni* infected mice, presumably through recruitment and differentiation from Ly6C^high^ monocytes. However, it was shown recently that basophil-derived IL-4 is required to induce Ly6C^high^ monocytes recruited to sites of allergic skin inflammation to acquire an AAM phenotype [36]. It is possible that innate sources of IL-4 drive AAM differentiation in models of acute inflammation, but that maintaining AAM during chronic *S. mansoni* infection requires an adaptive response from CD4^+^ T cells. Indeed, we have previously shown that CD4^+^ T cells are required for maintaining AAM during chronic helminth infection, but not in acute models of wound healing [31].

An unanswered question from this study is why the TH2 response induces AAM differentiation from monocytes in response to *S. mansoni* eggs, whereas during infection with a different helminth, *L. sigmodontis*, AAM predominantly arise through the proliferation of tissue-resident macrophages [10]. Why does IL-4 promote local macrophage proliferation and AAM differentiation under some circumstances, while promoting AAM differentiation from monocytes under other circumstances? Monocytes are recruited to sites of vascular damage and disease [37], so perhaps this difference is due in part to vascular damage caused by *S. mansoni* eggs becoming lodged in hepatic sinusoids. Another possibility is that the immune response to *S. mansoni* creates an inflammatory milieu that limits proliferation and favors monocyte recruitment. Although *S. mansoni* eggs drive a strong T_H_2 response, other cytokines such as IL-17 and IFN-ã are also produced [38], [39]. IFN-ã in particular has been shown to inhibit proliferation of several cell types, including bone marrow cells [40]. Thus, it is possible that the differences in the source of AAM following infection with *L. sigmodontis* and *S. mansoni* infection could be caused by differences in the cytokine milieu.

## Materials and Methods

### Mice and infections

*Cx_3_cr1^gfp/+^* mice were generously provided by Dr. Dan Littman (Skirball Institute, NYU) and were maintained by crossing homozygous *Cx_3_cr1* ^gfp/gfp^ mice to wild-type C57BL/6 mice. C57BL/6 mice were purchased from the Jackson Laboratory. Mice were infected percutaneously with 80–100 *S. mansoni* cerceriae harvested from infected *Biomphalaria glabrata* snails (Puerto Rican strain NMRI; Biomedical Research Institute). All mice were maintained under specific pathogen-free conditions at the New York University School of Medicine. This study was carried out in strict accordance with the recommendations in the Guide for the Care and Use of Laboratory Animals. All animal procedures were approved by the NYU Institutional Animal Care and Use Committee under protocol 090815. All surgery was performed under anesthesia, and all efforts were made to minimize suffering.

### CD4+ T cells depletions

Mice were depleted of CD4+ T cells using anti CD4 (BioXcell; Clone GK1.4). Both Uninfected and infected mice were given 0.25 mg of anti-CD4 i.p. every other day for one week. Infected mice were injected beginning at 5.5 weeks post-infection and analyzed at 6.5 weeks post infection.

### Tissue preparation

Livers were minced and incubated in 100 U/ml collagenase VIII (Sigma) and 150 ug/ml DNase I (Sigma) for 45 minutes at 37°C. Liver homogenates were dispersed through a 100 µm cell strainer (BD Biosciences) and leukocytes were enriched by density centrifugation over a 40/80% Percoll (GE Healthcare) gradient. Remaining RBCs were lysed with ACK lysis buffer (Quality Biologicals) and cells were washed and used for analysis.

### Flow cytometry

The following antibodies were used to phenotype liver leukocytes: aqua or blue amine-reactive viability dye (Invitrogen), CD11b eFluor450 (Ebioscience), PD-L2 PE (BD Bioscience, Biolegend), PD-L2-biotin followed by streptavidin PE-Alexa Fluor 610 (Invitrogen), Ly6C Alexa Fluor 700 (Clone Al-21, BD Bioscience), F4/80 PE-Cy7. CD3, B220, and DX5 conjugated to APC or CD3, B220, DX5, and Siglec F conjugated to PE were used to exclude non-myeloid cells and eosinophils from analysis. For *in vivo* CD45 staining [30], 1 ug of anti-CD45 Ab conjugated to either PE-Cy7 or PerCP-Cy5.5 (Biolegend) was injected intravenously 2 minutes prior to sacrificing mice. Cells were acquired on an LSR II (BD Biosciences) and analyzed using FlowJo software (Treestar).

### EdU labeling

Mice were injected intraperitoneally with 0.5 mg EdU (Invitrogen) 3 hours prior to sacrifice. Cells were surface stained, fixed, and permeabilized. Cells were stained intracellularly with anti-GFP AlexaFluor 647 (Biolegend) and then stained for EdU according to the manufacturer's instructions. Staining with PE-conjugated Abs was performed on permeabilized cells after the EdU reaction. A mouse that was not injected with EdU was used as a negative control (not shown).

### Quantitative real-time PCR

Quantitative RT-PCR was performed using the SYBR Green qPCR Mastermix (Applied Biosystems) as the detection dye. The comparative *C*t method was used to quantify the results obtained by qRT-PCR. Data were normalized to the housekeeping gene *Gapdh*. Primers were designed using Primer express V2.0 (Applied Biosystems) and synthesized by Integrated DNA technologies, sequences for the genes analyzed are as follows: GAPDH (sense) 5′-AATGGTGAAGGTCGGTGTGAAC-3′ and (antisense) 5′-AGGTCAATGAAGGGGTCGTTG-3′; CCR2 (sense) 5′-CAAATCAAAGGAAATGGAAGACAAT-3′ and (antisense) 5′- GCCCCTTCATCAAGCTCTTG-3′; Relmα (sense) 5′-CCCAGGATGCCAACTTTGAATAG-3′ and (antisense) 5′-AAGCCACAAGCACACCCAGTAG-3′; eGFP (sense) 5′- ACCACATGAAGCAGCACGACTTCT-3′ and (antisense) 5′- TCACCTTGATGCCGTTCTTCTGCT-3′; YM1 (sense) 5′-GCTCATTGTGGGATTTCCAGC-3′ and (antisense) 5′-CCTCAGTGGCTCCTTCATTCAG-3′

### Intravital imaging

*Cx_3_cr1^gfp/+^* mice were anesthetized with ketamine, xylazine, and acepromazine injected intramuscularly and were kept warm on a heating pad during surgery. Livers of anesthetized mice were exposed by carefully cutting through the skin and peritoneum just below the rib cage and gently coaxing out a lobe of the liver. Mice were then inverted onto a pre-warmed aluminum stage insert with a 2.5 cm window fitted with a 45×50 mm glass coverslip (Fisher Scientific). The liver was stabilized with gauze soaked in PBS to limit movement during imaging and to keep the liver moist. Mice were injected retro-orbitally with 250 µg of Hoechst 33342 to visualize nuclei and were transferred immediately to a heated chamber that maintains the microscope, objectives, mice, and stage at 37°C during imaging. In some experiments, mice were injected retro-orbitally with 10 µg of PE-conjugated anti-Ly6C (Clone HK1.4; Biolegend) or anti-GR-1 (clone Rb6-8C5; Biolegend). Images were acquired on a Leica SP2 AOBS inverted confocal microscope (20× HC PL APO 0.70 air or 40× HCX PL APO 1.25-0.75 oil objectives) with 405 nm, 488 nm, and 543 nm, 594 nm, and 633 nm excitation sources and detected using tunable filters. z stacks of a series of x-y planes were collected every 29.5–60 seconds with a step size of 2–4 µm and a total thickness of up to 20 µm. Images were collected using Leica LCS software.

### Image analysis

ImageJ64 (http://imagej.nih.gov/ij) was used to convert three-dimensional stacks into time series and create maximum projections of the z stacks. All images used to create time-lapse series were treated uniformly with a 0.7 pixel median filter. MTrackJ was then used to manually track single cells. Cells were only tracked if they were present for more than 5 frames. The mean speed, confinement ratios (mean displacement/track length) and arrest coefficients (% of time a cell crawls <2 um/min) and track duration were then calculated based on the tracking data as described [41]. Data from 3 individual mice was pooled for the uninfected group (n = 68). Data was pooled from 5 infected for eggs encapsulated in granulomas (n = 182) and 6 mice for eggs lodged in the blood vessels (n = 143).

### Monocyte transfers

Single cell suspensions of splenocytes from between 25–50 *Cx_3_cr1^gfp/+^* mice were stained with biotin-labeled anti-CD3 and anti-CD19 antibodies (eBioscience) and depleted of positive cells using anti-biotin microbeads and MACS depletion columns (Miltenyi Biotech). Monocytes were sorted from the remaining cells as described [42] by collecting single, live, lineage (CD3, B220, DX5, Ly6G, I-Ab, F4/80, Siglec F, CD11c) negative, CD11b+, GFP+, Ly6C^high^ or Ly6C^low^ cells. Each recipient mouse received either 2×10^5^ Ly6C^high^ or Ly6C^low^ monocytes intravenously. 24 hours after transfer, mice were injected i.v. with Pe-Cy7-conjugated anti-CD45 Ab and liver leukocytes were isolated and stained for CD11b, Ly6C, LIVE/DEAD viability, and PD-L2. A lineage negative gate including CD3, B220, DX5, and Siglec F was used to exclude cells from analysis.

### Statistical analyses

Data are displayed as mean ± SEM and were analyzed using One-way ANOVA followed by appropriate post-tests for multiple comparisons. Results from the CD4 depletion were compared using the Mann Whitney test.

## Supporting Information

Figure S1 **Sorting strategy for granuloma macrophages from the tissue and Ly6C^high^ and Ly6C^low^ monocytes from the blood.** Gating strategy used in Figure 5H to sort cells from liver tissue or blood at 8 weeks post-infection using *in vivo* CD45 staining (as described in the text) by injecting mice with anti-CD45 just prior to sacrifice. Liver leukocytes were isolated and single, live, lineage negative cells were gated on *in vivo* CD45+ (blood) and *in vivo* CD45− (tissue) populations. CD45+ (blood) cells were then gated on CX_3_CR1-GFP+CD11b+ cells, which were then sorted according to Ly6C expression as indicated. CD45− (tissue) cells were separated based on CD11b and CX_3_CR1-GFP expression to sort CX_3_CR1-GFP+CD11b+ granuloma macrophages and CD11b− CX_3_CR1-GFP− cells to use as a negative control.
(TIF)

Figure S2 **Sorting strategy for monocyte transfers.** Gating strategy to isolate CX3CR1-GFP+ Ly6C^high^ and Ly6C^low^ splenic monocytes used in experiments described in Figure 6 and Figure 7A. Splenocytes were first depleted of CD3+ and B220+ cells and then sorted as indicated.
(TIF)

Movie S1 **High magnification movie showing patrolling behavior of CX_3_CR1-GFP+ monocytes in the sinusoids of an uninfected liver.** Intravital confocal microscopy showing CX_3_CR1**-**GFP^+^ monocytes patrolling the hepatic sinusoids of an uninfected *Cx_3_cr1^gfp/+^* mouse. Host nuclei (blue) were visualized by injection of Hoechst 33342, CX_3_CR1-GFP^+^ cells are green, and tissue structure is visualized by auto-fluorescence (red). Tracks of crawling GFP+ cells are white and tracks of rapidly moving GFP+ cells are yellow. Z stacks were collected every 30 s and are shown at 6 frames per second.
(AVI)

Movie S2 **Crawling behavior of CX_3_CR1-GFP+ cells in a steady state uninfected liver.** Maximum projection time-lapse video collected by confocal microscopy showing GFP^+^ crawling monocytes in the hepatic sinusoids of an uninfected *Cx_3_cr1^gfp/+^* mouse. Host nuclei (blue) were visualized by injection of Hoechst 33342, CX_3_CR1-GFP^+^ cells are shown in green, and tissue structure is visualized by auto-fluorescence (red). Tracks of individual cells are white. Z stacks were collected every 30 s and are shown at 6 frames per second.
(AVI)

Movie S3 **Granuloma, showing motile round CX_3_CR1-GFP^+^ monocytes with stationary CX_3_CR1-GFP^+^ macrophages.** Maximum projection time-lapse video collected by confocal microscopy of the liver of a *Cx_3_cr1^gfp/+^* mouse 8 weeks post-infection showing an egg (red) in the tissue encased in a granuloma and surrounded by stationary GFP^+^ cells (green). Motile intravascular CX_3_CR1-GFP^+^ cells can be seen crawling near an egg lodged in the blood vessel and exposed to the vasculature. Tracks for individual cells are shown in white. Z stacks were collected every 30 s and are shown at 6 frames per second.
(AVI)

Movie S4 **Movement of CX_3_CR1-GFP^+^ monocytes around an egg encased in a fully developed granuloma.** Maximum projection of a time-lapse confocal microscopy video showing tracks (white) of single CX_3_CR1-GFP^+^ cells (green) crawling in the sinusoids around a fully developed granuloma. Many fast-moving CX_3_CR1-GFP^+^ cells can be seen, but were not tracked because they are in the imaging field for <5 frames. Z stacks were collected every 30 s and are shown at 6 frames per second.
(AVI)

Movie S5 **Movement of CX_3_CR1-GFP^+^ monocytes around an exposed egg in the liver.** Maximum projection of a time-lapse confocal microscopy video showing tracks (white) of single CX_3_CR1-GFP^+^ cells (green) crawling in the sinusoids around an exposed egg. Z stacks were collected every 30 s and are shown at 6 frames per second.
(AVI)

Movie S6 **Ly6C+ and Ly6C− GFP+ crawling cells near an egg lodged in the liver sinusoids.** Intravital confocal microscopy showing Ly6C+GFP+ and Ly6C-GFP+ cells crawling near an egg (red) lodged in the liver sinusoids at 8 weeks post-infection. Ly6C expression (red) was visualized by injecting mice i.v. with anti-Ly6C/Ly6G immediately prior to imaging. Ly6C+GFP+ (white tracks) and Ly6-GFP+ (yellow tracks) cells can be seen crawling in the sinusoids. Host nuclei (blue) were visualized by injection of Hoechst 33342, CX_3_CR1-GFP^+^ cells are shown in green, and tissue structure is visualized by auto-fluorescence (red). Z stacks were collected every 30 s and are shown at 6 frames per second.
(AVI)
