# Supplementary figures and images for: Ly6Chigh Monocytes Become Alternatively Activated Macrophages in Schistosome Granulomas with Help from CD4+ Cells
Source: PLoS Pathog. 2014 Jun 26;10(6):e1004080. doi: 10.1371/journal.ppat.1004080 (PMC4072804; doi:10.1371/journal.ppat.1004080)

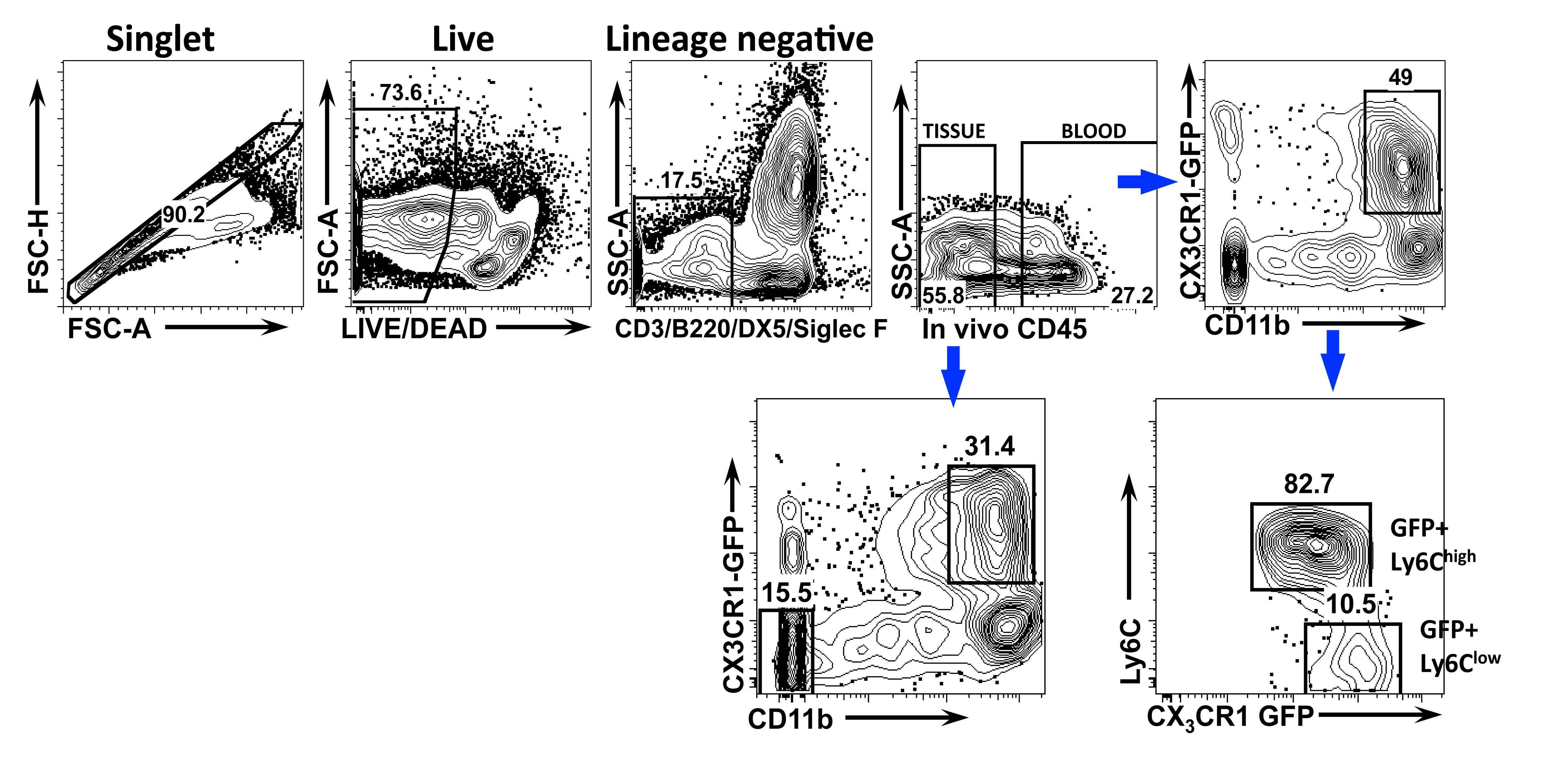

Supplement: Figure S1 — Sorting strategy for granuloma macrophages from the tissue and Ly6Chigh and Ly6Clow monocytes from the blood. Gating strategy used in Figure 5H to sort cells from liver tissue or blood at 8 weeks post-infection using in vivo CD45 staining (as described in the text) by injecting mice with anti-CD45 just prior to sacrifice. Liver leukocytes were isolated and single, live, lineage negative cells were gated on in vivo CD45+ (blood) and in vivo CD45− (tissue) populations. CD45+ (blood) cells were then gated on CX3CR1-GFP+CD11b+ cells, which were then sorted according to Ly6C expression as indicated. CD45− (tissue) cells were separated based on CD11b and CX3CR1-GFP expression to sort CX3CR1-GFP+CD11b+ granuloma macrophages and CD11b− CX3CR1-GFP− cells to use as a negative control. (TIF) [file ppat.1004080.s001.tif]

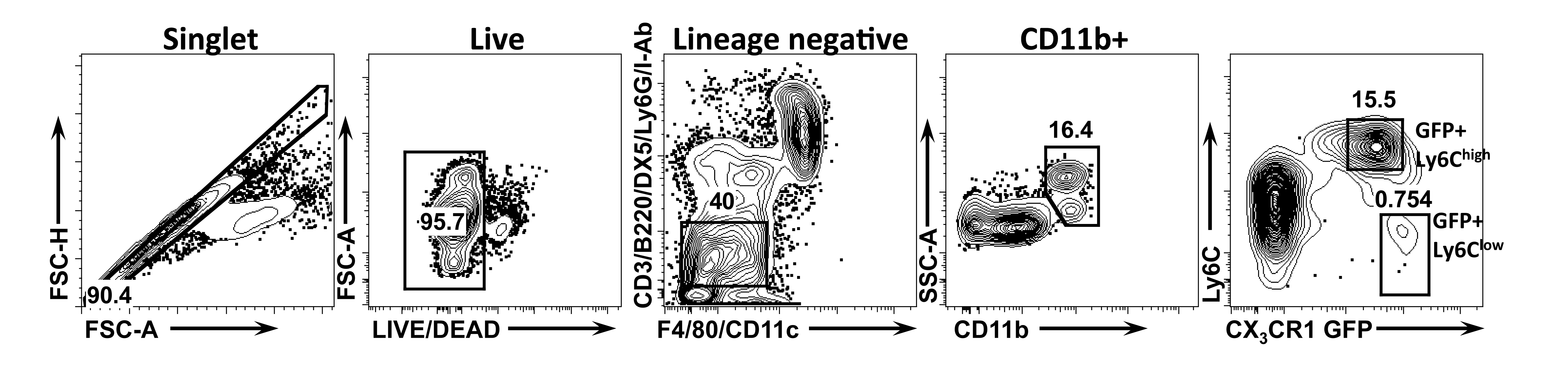

Supplement: Figure S2 — Sorting strategy for monocyte transfers. Gating strategy to isolate CX3CR1-GFP+ Ly6Chigh and Ly6Clow splenic monocytes used in experiments described in Figure 6 and Figure 7A. Splenocytes were first depleted of CD3+ and B220+ cells and then sorted as indicated. (TIF) [file ppat.1004080.s002.tif]
